# Viruses and Protists Induced-mortality of Prokaryotes around the Antarctic Peninsula during the Austral Summer

**DOI:** 10.3389/fmicb.2017.00241

**Published:** 2017-03-02

**Authors:** Dolors Vaqué, Julia A. Boras, Francesc Torrent-Llagostera, Susana Agustí, Jesús M. Arrieta, Elena Lara, Yaiza M. Castillo, Carlos M. Duarte, Maria M. Sala

**Affiliations:** ^1^Institut de Ciències del Mar (CSIC), Consejo Superior de Investigaciones CientíficasBarcelona, Spain; ^2^King Abdullah University of Sciences and TechnologyThuwal, Saudi Arabia; ^3^Institute of Marine Sciences (CNR-ISMAR), National Research CouncilVenezia, Italy

**Keywords:** viruses, prokaryotes, protists, lysis, lysogeny, mortality, temperature, Antarctic waters

## Abstract

During the Austral summer 2009 we studied three areas surrounding the Antarctic Peninsula: the Bellingshausen Sea, the Bransfield Strait and the Weddell Sea. We aimed to investigate, whether viruses or protists were the main agents inducing prokaryotic mortality rates, and the sensitivity to temperature of prokaryotic heterotrophic production and mortality based on the activation energy (Ea) for each process. Seawater samples were taken at seven depths (0.1–100 m) to quantify viruses, prokaryotes and protists abundances, and heterotrophic prokaryotic production (PHP). Viral lytic production, lysogeny, and mortality rates of prokaryotes due to viruses and protists were estimated at surface (0.1–1 m) and at the Deep Fluorescence Maximum (DFM, 12–55 m) at eight representative stations of the three areas. The average viral lytic production ranged from 1.0 ± 0.3 × 10^7^ viruses ml^−1^ d^−1^ in the Bellingshausen Sea to1.3 ± 0.7 × 10^7^ viruses ml^−1^ d^−1^ in the Bransfield Strait, while lysogeny, when detectable, recorded the lowest value in the Bellingshausen Sea (0.05 ± 0.05 × 10^7^ viruses ml^−1^ d^−1^) and the highest in the Weddell Sea (4.3 ± 3.5 × 10^7^ viruses ml^−1^ d^−1^). Average mortality rates due to viruses ranged from 9.7 ± 6.1 × 10^4^ cells ml^−1^ d^−1^ in the Weddell Sea to 14.3 ± 4.0 × 10^4^ cells ml^−1^ d^−1^ in the Bellingshausen Sea, and were higher than averaged grazing rates in the Weddell Sea (5.9 ± 1.1 × 10^4^ cells ml^−1^ d^−1^) and in the Bellingshausen Sea (6.8 ± 0.9 × 10^4^ cells ml^−1^ d^−1^). The highest impact on prokaryotes by viruses and main differences between viral and protists activities were observed in surface samples: 17.8 ± 6.8 × 10^4^ cells ml^−1^ d^−1^ and 6.5 ± 3.9 × 10^4^ cells ml^−1^ d^−1^ in the Weddell Sea; 22.1 ± 9.6 × 10^4^ cells ml^−1^ d^−1^ and 11.6 ± 1.4 × 10^4^ cells ml^−1^ d^−1^ in the Bransfield Strait; and 16.1 ± 5.7 × 10^4^ cells ml^−1^ d^−1^ and 7.9 ± 2.6 × 10^4^ cells ml^−1^ d^−1^ in the Bellingshausen Sea, respectively. Furthermore, the rate of lysed cells and PHP showed higher sensitivity to temperature than grazing rates by protists. We conclude that viruses were more important mortality agents than protists mainly in surface waters and that viral activity has a higher sensitivity to temperature than grazing rates. This suggests a reduction of the carbon transferred through the microbial food-web that could have implications in the biogeochemical cycles in a future warmer ocean scenario.

## Introduction

To understand the functioning of the biogeochemical carbon cycles in extreme marine systems, such as the Antarctic Ocean, it is essential to elucidate the relation between the prokaryotic members of the community and their predators, protists and viruses, since their impact in the carbon cycles are different. While protists channel prokaryotic carbon to higher trophic levels through the grazing process, viruses return dissolved and small particulate prokaryotic carbon forms from the lysed cells (Wilhelm and Suttle, 1999) to the water column (the viral shunt), and may modify the efficiency of the carbon pump (Suttle, 2007). Indeed, viruses contribute to generate new substrates for prokaryotes, increasing respiration (Eissler et al., 2003), nutrients regeneration (Gobler et al., 1997), and being crucial for Fe regeneration, an important trace element for phytoplankton growth (Strzepek et al., 2005; Evans and Brussaard, 2012).

The ecology of prokaryotes and protists in Antarctic waters has been well studied since the 1980's (e.g., Karl et al., 1991; Kuparinen and Bjørsen, 1992; Leaky et al., 1996; Vaqué et al., 2002a,b; Christaki et al., 2008) but less is known on the role of viruses (Danovaro et al., 2011). The few studies focused on viral infection of prokaryotes were carried out mainly in waters adjacent to the Antarctic Peninsula (Guixa-Boixereu et al., 2002), in coastal regions of Antarctica (Pearce et al., 2007), the Weddell Sea and Polar Frontal zones, and in the Southern Ocean including Sub-Antarctic areas (Bonilla-Findji et al., 2008; Evans et al., 2009; Evans and Brussaard, 2012; Malits et al., 2014). All those studies point toward a high viral lytic production and suggest a major role of viruses in prokaryotic mortality. However, if we aim at elucidating which is the main source of prokaryotic mortality both viral production and protistan grazing should be measured simultaneously, what has been done only in two studies: Guixa-Boixereu et al. (2002) in surface samples around the Antarctic Peninsula and Christaki et al. (2014) in Sub-Antarctic waters. In both cases they reported that mortality rates caused by viruses were higher (at least in some periodes of the year) than those caused by protists, suggesting that the viral shunt would drive the destiny of the prokaryotic carbon in those regions.

The impact of viruses and protists on prokaryotes reported in Guixa-Boixereu et al. (2002) during the Austral summer in the Bellingshausen Sea and the Gerlache Strait was very high, removing ≥ 50% d^−1^ of its biomass and more than 100% d^−1^ of its heterotrophic production, which suggests that prokaryotes were top-down controlled. Then, to maintain a sustainable prokaryotic heterotrophic production and biomass, it would imply that DOC should not be a limiting factor for prokaryotes to grow. Indeed, Pedrós-Alió et al. (2002), point out that during the Austral Summer in the Antarctic Ocean prokaryotic production was not constrained by DOC.

Finally, according to the available literature, values of prokaryotic production and mortality rates in Antarctic waters do not differ much from those found in temperate systems (e.g., Guixa-Boixereu et al., 2002; Boras et al., 2009), although microbial processes in the Antarctic occur at very low temperatures. However, an increase of temperature in marine ecosystems, including Polar Regions, enhances heterotrophic prokaryotic production and mortality rates (Vázquez-Domínguez et al., 2007; Vaqué et al., 2009; Lara et al., 2013). Sarmento et al. (2010) compiled data of prokaryotic production and grazing rates from temperate systems to Antarctic waters, and have shown a higher sensitivity to temperature for prokaryotic production than for mortality rates. The same was found by Maranger et al. (2015), who included also the rate of lysed prokaryotes for the Arctic Ocean into the dataset. It is then important to elucidate the sensitivity of these processes in the Antarctic Ocean, in which this information is lacking, since they may have consequences in the fate of the microbial carbon in a warmer ocean.

In the present study, we aimed to test the following working hypotheses: (a) since high viral production rates were detected previously in polar systems, the mortality caused by viruses should be at least as important or higher than the protistan impact in Antarctic waters; (b) taking into account that DOC is not a limiting factor for prokaryotic production during the Austral summer, we expected that prokaryotes are top-down controlled at this time of the year; (c) prokaryotic production, viral infection and grazing by protists would show different sensitivity to temperature changes as suggested by study in the Arctic Ocean. To verify these hypotheses we evaluated prokaryotic losses due to viruses and protists in three different areas around the Antarctic Peninsula (the Weddell Sea, the Bransfield Strait and the Bellingshausen Sea). We elucidated the impact of predators on the prokaryotic community (top-down control) and we assessed the sensitivity of the microbial processes (prokaryotic production and mortalities) to the different temperatures recorded at the three visited areas. This study will contribute to add new information on the fate of prokaryotic carbon within the microbial food-web, and how the impact of temperature on heterotrophic prokaryotes production and losses, which will affect the biogeochemical cycles, could generate new hypothesis in a warmer ocean scenario.

## Materials and methods

### Sampling sites

**Figure 1:**
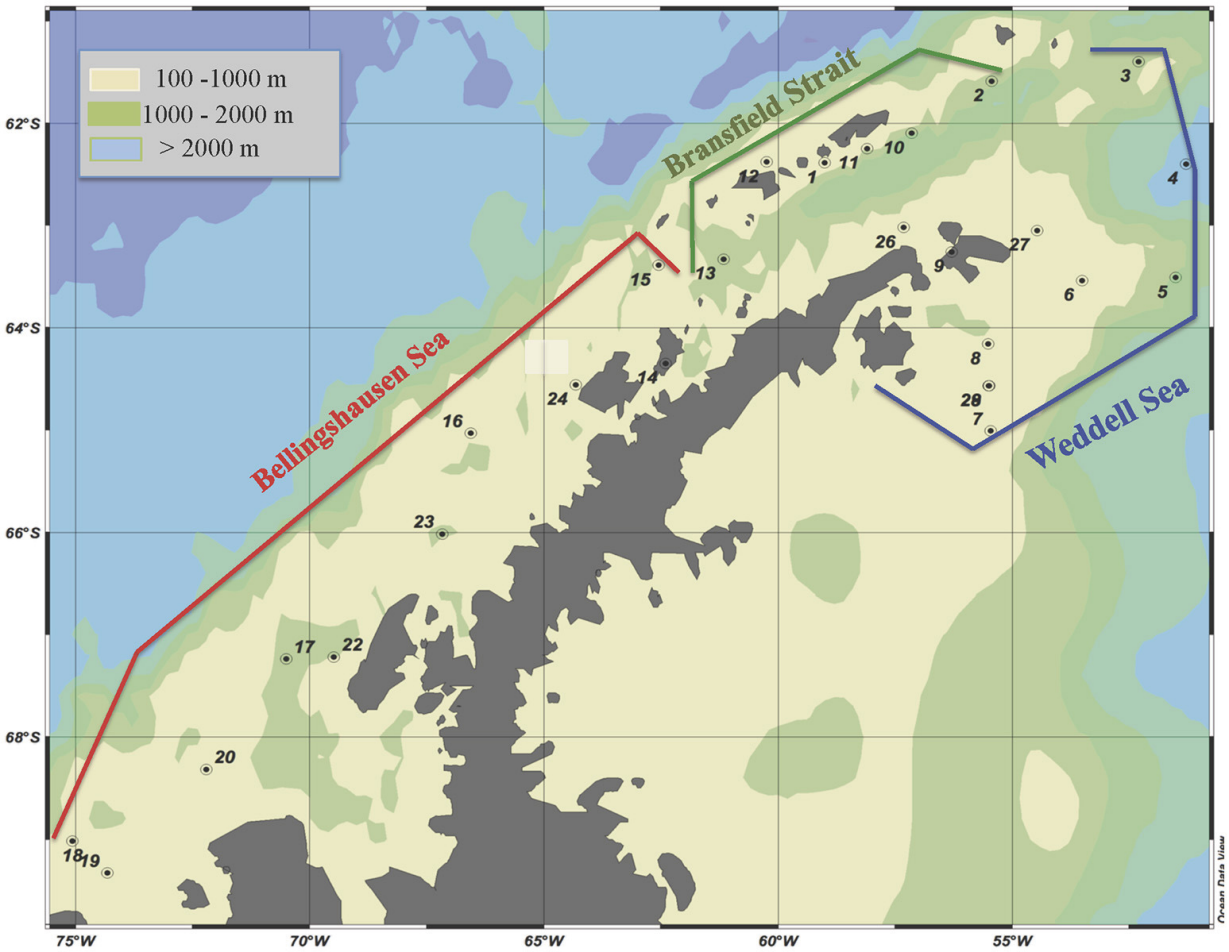
**Location of the visited stations in each delimited area**. Data on heterotrophic microorganisms is not available for station 14. Ocean data view is used for mapping (Schlitzer, 2017).

A cruise was carried out on board of the R/V BIO Hespérides from January 28 to February 25, 2009, around the Antarctic Peninsula. The studied area and sampling sites are shown in Figure 1 and Table 1SM. We sampled three representative Antarctic areas: (1) the Weddell Sea (WS); (2) the western basin of the Bransfield Strait (BrS); and (3) the Bellinghausen Sea (BeS). These areas were characterized according to their location, physicochemical properties as well as water circulation (Garcia et al., 2002; Gòmis et al., 2002; Hellmer et al., 2011).

### Physicochemical variables and microbiological biomasses

Profiles of salinity, temperature, and units of fluorescence (as a proxy of chlorophyll *a* concentration) were obtained using a CTD EG&G model MkIIIC WOCE between 1 and 100 m depth (Table 1SM). Samples for the microbiological parameters were taken from 0.1 or 1 m to 100 m, at seven depths: from two to three above the DFM (deep fluorescence maximum), one at the DFM, and from two to three below the DFM (Table 1SM). Samples from 0.1 m were collected directly from a rubber boat, and for the other six depths with 12 L Niskin bottles attached to a rossette sampler system. Due to weather conditions, no measurements were done for 0.1 m at stations 4, 11, 15, 23, and 27. Viruses and prokaryotes abundances were measured at all depths and at all stations (Table 1SM). Subsamples for viral abundances (2 ml) were fixed with glutaraldehyde (0.5% final concentration), quick frozen in liquid nitrogen as described by Brussaard (2004) and stored at −80°C. Subsamples for prokaryote abundances (2 ml) were fixed with paraformaldehyde (1% final concentration). Virus samples were stained with SYBR- GreenI and analysed as described in Brussaard (2004). Prokaryote samples were stained with SYTO13 according to the described in Gasol and del Giorgio (2000), and were run using 0.92-μm yellow-green latex beads as an internal standard. Viral and prokaryotes counts were made on a FACSCalibur (Becton & Dickinson) flow cytometer, back in the Institut de Ciències del Mar (ICM) lab. Virus-prokaryote ratio VPR was calculated dividing the *in situ* viral abundance by *in situ* prokaryotic abundance. Viral biomass was calculated using the factor 1 × 10^−16^ g C virus ^−1^ described by Børsheim et al. (1990), and prokaryotic biomass was estimated using the carbon-to-volume relationship equation derived by Norland (1993) from the data of Simon and Azam (1989): pg C cell^−1^ = 0.12 pg x (μm^3^ cell^−1^)^0.7^. We assumed an average prokaryote cell volume of 0.047 μm^3^ cell^−1^ measured in similar Antarctic waters (Vaqué et al., 2002a, 2009). Heterotrophic (HF) and phototrophic (PF) pico/nanoflagellates abundances were measured at eight selected stations, at depths where prokaryotic mortality rates were recorded (Table 1SM). They were counted by epifluorescence microscopy (Olympus BX40-102/E at 1,000X) back to the ICM lab. Subsamples (50 ml) were fixed with glutaraldehyde (1% final concentration), filtered through 0.6 μm black polycarbonate filters and stained with DAPI (4,6-diamidino 2-phenylindole) at a final concentration of 5 μg ml^−1^ (Sieracki et al., 1985). PF could be distinguished from HF under blue light, as the presence of plastidic structures with red fluorescence in PF could be observed. At least 50–100 cells for HF and PF were counted per filter. They were grouped into 2 size classes: ≤ 5 μm and >5 μm. Biomass of HF and PF was calculated using a volume to carbon ratio of 0.22 pg C μm^−3^ (Børsheim and Bratbak, 1987). Cell volumes were estimated assuming spherical shapes and a diameter of 3 μm, taking into account that 95% of HF and PF had a diameter smaller than 5 μm. Microphytoplankton samples were collected at all stations at the surface (1 m) and at the DFM. 30 ml of each sample was filtered onto 2 μm pore-size black polycarbonate filters, fixed with glutaraldehyde (1% final concentration) and stored frozen at −80°C until counting under an epifluorescence microscope (Zeiss Axioplan Imaging) back to the IMEDEA lab. Two majors groups, dinoflagellates and diatoms, were identified. The average cell volume for each phytoplankton group identified during the study was computed using the geometrical approximation of their forms, and the biovolume (μm^3^ L^−1^) of the different phytoplankton groups in each sample was calculated as the product of the cell density (cell L^−1^) multiplied by average cell volume (μm^3^ cell^−1^).

### Prokaryotic heterotrophic production

Prokaryotic heterotrophic production (PHP) was determined at all stations and depths (Table 1SM). It was estimated by the radioactive ^3^H-leucine incorporation technique (Kirchman et al., 1985), with the modifications established for the use of microcentrifuge vials (Smith and Azam, 1992). The vials were counted in a Beckman scintillation counter on board. PHP was calculated according to the equation:

$$\begin{array}{l} \text{PHP}=\text{LeuxCF }(\mu \text{gC}{\text{L}}^{-1}{\text{ day}}^{-1}), \end{array}$$

where Leu is the ^3^H-leucine incorporation (pmol l^−1^ h^−1^) and CF is the conversion factor (1.5 kg C mol Leu^−1^, Kirchman, 1992).

### Prokaryotic mortality due to protists

Grazing rates on prokaryotes by protists (GZ) were evaluated at eight representative stations (Table 1SM) and at two selected depths: surface (0.1 or 1 m) and DFM, using the fluorescently labeled bacteria (FLB) disappearance method (Vázquez-Domínguez et al., 1999). The FLB used in this study were prepared with a culture of *Brevundimonas diminuta* provided by the Spanish Type Culture Collection (www.uv.es/cect). The size of these bacteria (0.064 μm^3^ cell^−1^) is comparable to the natural marine prokaryotes recorded in Antarctic waters (0.040–0.070 μm^3^ cell^−1^) (Vaqué et al., 2002a, 2009). The FLB work solutions were prepared in the ICM lab and stored frozen at −20°C until use. Grazing rate experiments were run in 2-L polycarbonate bottles, in duplicates (1 L of seawater each) plus one prokaryote-free control (1 L of seawater filtered with a 30 kDa cartridge). All bottles (control and duplicates) were inoculated with FLB at 20% of the *in situ* prokaryote concentration (assessed for this purpose on the on board epifluorescence microscope, BX40-102/E at 1,000X, with the previous staining with DAPI). The added FLB oscillated between 4.1 × 10^4^ and 1.6 × 10^5^ cells ml^−1^ (final concentration). Bottles were incubated in a thermostatic chamber that simulated the *in situ* temperature, in the dark, for 48 h. Samples for prokaryotes and FLB abundances were taken at the beginning and at the end of the incubations. The abundances were determined with epifluorescence microscopy (Olympus BX40-102/E; 1,000 X magnification) back to the ICM lab, after filtering the sample (20 ml) through 0.2-μm black polycarbonate filters and staining with DAPI at a final concentration of 5 μg ml^−1^ (Sieracki et al., 1985). Natural prokaryotes were identified by their blue fluorescence when excited with UV radiation, while FLB were identified by their yellow-green fluorescence when excited with blue light. Protists grazing rates were calculated following the equations of Salat and Marrasé (1994). For details see Boras et al. (2010).

### Viral production and prokaryotic mortality due to viruses

Viral production (VP) and prokaryotic losses due to viruses (rate of lysed cells, RLC) were measured at the same stations and depths as GZ (Table 1SM). VP was determined following the virus-reduction approach (Weinbauer et al., 2002). This method distinguishes between the production of lytic (VP_L_), and lysogenic phages (VP_Lyso_), by inducing lysis with mitomycin C. To perform the VP measurements, one liter of seawater was filtered tangentially on the VIVAFlow 200 cartridges to obtain the prokaryote concentrate (40 ml) and the virus-free water as described in details in Boras et al. (2010). A mixture of virus-free water (160 ml) and prokaryote concentrate (40 ml) was prepared and distributed into four sterile 50-ml falcon plastic tubes. Two of the tubes were kept as controls to measure viral lytic production, while mitomycin C (Sigma) was added to the other two tubes as the inducing agent of the lytic cycle in prophages (1 μg ml^−1^ final concentration). The tubes were incubated in a thermostatic chamber simulating *in situ* temperature, in the dark for 12 h. Samples for viral and prokaryotic abundances were collected at time zero and every 4 h of incubation, fixed with glutaraldehyde (0.5% final concentration) and stored as described before for viruses abundance. Viruses and prokaryotes from viral production incubations were counted by flow cytometry, as described above, back to the lab in the ICM. Calculations of viral lytic and lysogenic production were made according to Weinbauer et al. (2002). As part of the prokaryotes is lost during the prokaryotic concentration process, the VP_L_ and VP_Lyso_ were multiplied by the prokaryote correction factor (Winget et al., 2005, from 1.7 to 10 in our study) to enable the comparison of the VP from different incubations. The number of viruses released by a prokaryote cell (burst size, BS) was estimated from viral production incubations, as in Jiang and Paul (1996) and Boras et al. (2009), and ranged from 12 to 126 viruses per cell.

The RLC was calculated by dividing VP_L_ by BS as is described in Guixa-Boixereu (1997):

$$\begin{array}{l} \text{RLC }\left({\text{cells lysed ml}}^{-1}{\text{d}}^{-1}\right)={\text{VP}}_{\text{L}}/\text{BS} \end{array}$$

The RLC was used also to calculate the % of the prokaryotic standing stock that was lysed by viruses (PSS_RLC_):

$$\begin{array}{l} \%{\text{PSS}}_{\text{RLC}}\;\left({\text{d}}^{-1}\right)=\text{RLC}\times 100/\text{PSS} \end{array}$$

Where PSS is the prokaryotic standing stock abundance.

### Sensitivity of microbial processes to temperature

The temperature sensitivities of the different microbial processes were obtained by estimating the activation energy (Ea) of each one of them, using the Boltzmann-Arrhenius model:

$$\begin{array}{l} \text{B}={\text{B}}_{0}\times {\text{e}}^{\left(\text{Ea}/\text{kT}\right)} \end{array}$$

where, B is the basal metabolic rate (i.e., of PHP, GZ, and RLC) and B_0_ is a normalization constant independent of body-size and temperature.

The term e^(Ea/kT)^ is the Boltzmann factor that describes the temperature (T, in Kelvin degrees) dependence of a metabolic rate, where k is the Boltzmann's constant (8.62 × 10^−5^ eV k^−1^) and Ea the activation energy of the given rate (West et al., 1997; Brown et al., 2004).

This equation can also be written as:

$$\begin{array}{l} \text{ln B}={\text{ln B}}_{0}+\text{Ea}\times 1/\text{kT} \end{array}$$

Ea is the slope; obtained when plotting lnB against 1/kT. The steeper is the slope the greater is the sensitivity to temperature changes.

### Statistical analyses

All data except temperature and salinity were log transformed. To estimate differences of physicochemical and biological parameters among the Antarctic areas we carried out one-way ANOVA analyses and the subsequent *post-hoc* Tukey test. Student's *t*-test was applied to test differences of mortality rates between surface and DFM, and between grazing rates and lysed rates for each station and depth. Regression analyses were carried out between different microbiological parameters. All statistical analyses were performed with the JMP 8.0 program.

## Results

### Physicochemical and biological parameters

The water column was always stratified showing a DFM between 20 and 50 m in the Weddell Sea, 15 and 30 m in the Bransfield Strait, and 12 and 55 m in the Bellingshausen Sea (Table 1SM). ANOVA analyses revealed that in the Weddell Sea temperature and salinity had significant lower values (−0.49°C ± 0.09, 27.86 ± 0.07, respectively) (Table 1 and Table 2SM) than the other two areas. Units of Fluorescence (UF, a proxy of phytoplankton biomass), reached the highest value in station 7 (the Weddell Sea, 14.60), but significant differences were only found between the Bransfield Strait (1.66 ± 0.18) and the Bellingshausen Sea (1.10 ± 0.15, Table 1 and Table 2SM). However, the phototrophic pico/nanoflagellates (PF) did not show significant differences among the three areas (Table 1). Microphytoplankton dominated the biomass in the Weddell Sea (66% of total biovolume), and phototrophic nanoflagellates dominated in the other two zones, the Bellingshausen (56% of total biovolume) and the Bransfield (66% of total) areas. Centric diatoms prevailed along the cruise, while pennate forms, mainly represented by *Pseudo-Nitzschia Seriata, Pseudo-Nitzschia Delicatissima* and *Navicula* sp., displayed similar biovolumes. We detected in the Weddell Sea an increase in the biovolume of centric diatoms, being mostly represented by *Thalassiosira sp*. while *Corethron sp*. and *Coscinodiscus sp*. were predominant forms in the Bransfield and the Bellingshausen zones.

**Table 1:** **Depth-averaged and minimum and maximum values for the three areas, ***n***: number of data**.

**Variables**	**Weddell sea**		**Bransfield strait**		**Bellingshausen sea**	
	**Average**	***n***	**Average**	***n***	**Average**	***n***
	**Min-max**		**Min-max**		**Min-max**	
Temperature (°C)	**−0.49**	59	**1.30**	42	**1.20**	45
	−1.79–0.14		−0.75–2.84		−1.50–3.24	
Salinity	**27.86**	45	**29.54**	33	**28.95**	45
	27.11–28.88		28.01–30.37		26.15–30.62	
Units of Fluorescence (UF)	**2.07**	45	**1.66**	43	**1.23**	39
	0.24–14.60		0.21–3.12		0.20–4.90	
Prokaryotes abundance	**3.08**	55	**3.67**	43	**4.84**	55
(10^5^ cells ml^−1^)	1.33–6.34		1.67–6.49		1.74–11.40	
Viruses abundance	**4.11**	61	**5.69**	43	**11.30**	55
(10^6^ cells ml^−1^)	0.17–22.1		0.72–20.30		0.16–83.10	
VPR	**15.00**	55	**18.04**	43	**33.54**	55
	0.56–63.79		1.12–88.14		0.81–332.21	
HF	**1.75**	14	**1.19**	21	**0.97**	21
(103 cells ml^−1^)	0.51–5.09		0.29–2.62		0.17–1.90	
PF	**2.88*****a***	14	**3.25*****a***	21	**3.78**	21
(103 cells ml^−1^)	1.12–5.29		0.51–6.33		0.48–10.57	
PHP	**1.94*****a***	54	**1.32*****a***	47	**1.52**	55
(10^5^ cells ml^−1^ d^−1^)	0.05–6.83		0.06–3.24		0.05–2.84	
PHP	**2.72**	54	**1.85**	47	**2.13**	55
(μg C L^−1^ d^−1^)	0.07–9.53		0.09–4.54		0.07–3.98	
VP_L_	**1.23**	4	**1.28**	6	**1.00**	6
(10^7^ viruses ml^−1^ d^−1^)	0.19–3.47		0.10–4.43		0.22–2.31	
VP_L_ (Surface & DFM)	**2.26 & 0.21**	2-2	**2.27 & 0.29**	3-3	**0.72 & 1.29**	3-3
(10^7^ viruses ml^−1^ d^−1^)	1.1–3.5; 0.2–0.2		0.7–4.4; 0.1–0.6		0.3–1.6; 0.2–2.3	
VP_Lyso_	**4.32**	4	**0.11**	6	**0.05**	6
(10^7^ viruses ml^−1^ d^−1^)	nd (2)–16.50		nd (1)–0.25		nd (5)–0.30	
VP_Lyso_ (Surface & DFM)	**8.25 & 0.40**	2-2	**0.16 & 0.06**	3-3	**nd & 0.10**	3-3
(10^7^ viruses ml^−1^ d^−1^)	nd(1)–16.5; nd(1)–0.8		nd(1)– 0.3; 0.03–0.1		nd(3); nd(2)–0.3	
GZ	**5.87**	4	**10.10**	6	**6.79**	6
(10^4^ cells ml^−1^ d^−1^)	1.99–8.98		7.81–14.90		1.84–12.4	
GZ (Surface &DFM)	**6.50 & 5.23**	2-2	**11.60 & 8.53**	3-3	**7.91 & 5.67**	3-3
(10^4^ cells ml^−1^ d^−1^)	6.0–7.1; 2.0–8.5		9.6–14.9; 7.8–8.9		12.4–1.84; 4.0–7.7	
RLC	**9.72**	4	**12.30**	6	**14.30**	6
(10^4^ cells ml^−1^ d^−1^)	1.47–27.40		1.59–45.50		1.70–26.20	
RLC (Surface & DFM)	**17.80 & 1.62**	2-2	**22.10 & 2.59**	3-3	**16.10 & 12.40**	3-3
(10^4^ cells ml^−1^ d^−1^)	8.2–27.4; 1.7–1.7		7.5–45.5; 1.6–4.5		2.9–26.2; 1.7–18.2	
%PSS_GZ_ (d^−1^)	**20.56**	4	**29.10**	6	**15.06**	6
	4.46–35.42		18.25–37.77		1.68–30.48	
%PSS_GZ_ (d^−1^) (Surface & DFM)	**22.85 & 18.27**	2-2	**32.98 & 25.22**	3–3	**20.20 & 9.93**	3-3
	10.4–35.3; 4.5–32.1		26.8–37.7; 18.3–30.6		1.7–30.5; 6.8–15.6	
%PSS_RLC_ (d^−1^)	**24.74**	4	**33.92**	6	**28.44**	6
	3.30–47.75		5.07–123.52		2.76–78.17	
%PSS_RLC_ (d^−1^) (Surface& DFM)	**44.47 & 5.00**	2-2	**60.83 & 7.02**	3-3	**32.87 & 24.00**	3-3
	41.2–47.8; 3.3–6.7		27.7–123.5; 5.1–10.5		2.8–78.2; 3.2–53.	

*VPR (virus-prokaryote ratio), HF and PF (heterotrophic and phototrophic pico/nanoflagellates), PHP (prokaryote heterotrophic production), VP_L_ (viral lytic production), VP_Lyso_ (lysogeny), GZ (grazing rates on bacteria), RLC (rates of lysed prokaryotic cells), %PSS (percentage of prokaryotes removed by protists or by viruses). In gray averages of viral and protists processes in surface and in the DFM. nd: no detected. Number in parenthesis: cases where VP_Lyso_ was not detected. In bold averaged values*.

**Figure 2:**
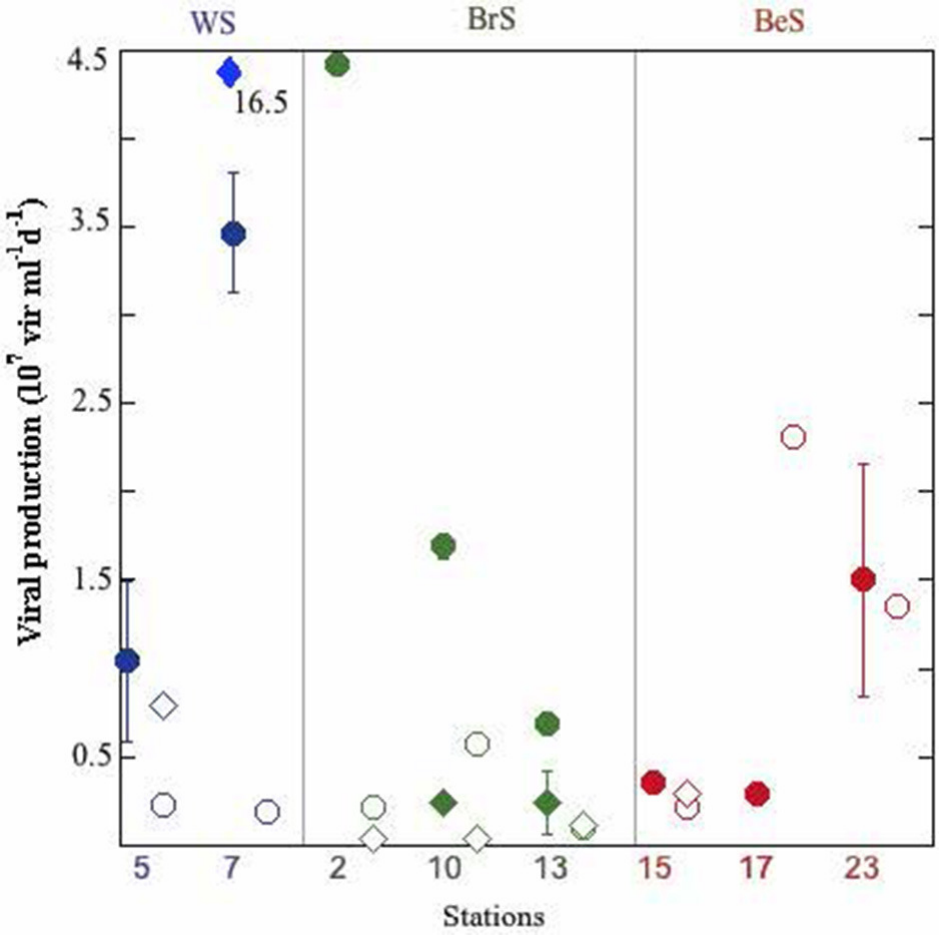
**Viral lytic and lysogenic production in each station and depth**. Full circles: lytic production at surface; empty circles: lytic production at DFM; full diamonds: lysogenic production at surface; empty diamonds: lysogenic production at DFM. Outlier value for lysogeny at surface of station 7 is indicated.

In the Weddell Sea both, abundances of prokaryotes (3.08 ± 0.16 × 10^5^ cell ml^−1^) and viruses (4.11 ± 0.73 × 10^6^ virus ml^−1^) showed significantly lower values (Table 1, Table 2SM) than in the other two areas. In contrast, heterotrophic pico/nanoflagellates (HF) displayed significantly higher values for the Weddell Sea (1.75 ± 0.34 × 10^3^ cell ml^−1^) than the Bellingshausen Sea (0.97 ± 0.01 × 10^3^ cell ml^−1^) (Table 1 and Table 2SM). Prokaryotic heterotrophic production (PHP) and viral lytic production (VP_L_) did not show differences among the three areas (ANOVA, *p* > 0.05), although the highest values for PHP were registered in the Weddell Sea (6.83 × 10^5^ cells ml^−1^ d^−1^), and for VP_L_ in the Bransfield Strait (4.43 × 10^7^ viruses ml^−1^ d^−1^) (Table 1, Figure 2). Lysogeny (VP_Lyso_) in the Weddell Sea was registered twice, at the DFM of station 5 (78% of VP) and at the surface of station 7 (83% of VP). In the Bransfield Strait it was found at all sampled stations at surface and DFM, except at surface of station 2. In the Bellingshausen Sea was only detected once, at station 15 at DFM (50% of total VP) (Table 1SM, Figure 2). The average percentage of prokaryotes consumed by grazers showed significantly higher averaged value in the Bransfield Strait (29.10 ± 2.87% d^−1^) in respect to the Bellingshausen Sea (15.06 ± 4.91% d^−1^), while the averaged values of the lysed prokaryote cells due to viruses was higher, although not significantly, in the Bransfield Strait (32.92 ± 18.50% d^−1^) than the other two areas (Table 1, Table 1SM).

When pooling all data for the three areas, we observed that PHP was significantly related with UF (indicator of phytoplankton biomass), and with HF and viral abundances (main prokaryote mortality agents, Figure 1SM, Table 2). However, when considering each area separately, the tightest relationship between PHP and UF was obtained in the Weddell Sea, where UF explained a 75% of PHP variability. Furthermore, HF and PHP achieved the strongest relationships in the Bransfield Strait, where PHP explained a 69% of the HF abundance variability (Table 2). Finally, the strongest relationship between viral abundance and PHP was found in the Bellingshausen Sea, where PHP explained 40% of the variability of viral abundance (Table 2).

**Table 2:** **Regression equations (y = a + bx) between: prokaryotic heterotrophic production (PHP, μg C L^**−1**^ d^**−1**^) and Units of Fluorescence (UF); heterotrophic pico/nanoflagellate (HF, cells ml^**−1**^) and PHP; viral abundance (VA, virus ml^**−1**^) and PHP**.

**Site**	**Dp Var**	**Indp Var**	**Intersect**	**Slope**	***n***	***R*****^2^**	***p***
All	logPHP	logUF	0.08	0.63	38	0.383	<0.001
WS	logPHP	logUF	0.16	0.56	10	0.750	<0.001
BrS	logPHP	logUF	−0.10	1.03	14	0.673	<0.001
BeS	logPHP	logUF	0.16	0.59	14	0.119	ns
All	logHF	logPHP	2.90	0.54	55	0.545	<0.0001
WS	logHF	logPHP	3.05	0.33	14	0.250	<0.05
BrS	logHF	logPHP	2.81	0.57	20	0.687	<0.0001
BeS	logHF	logPHP	2.92	0.58	21	0.621	<0.0001
All	logVA	logPHP	6.41	0.45	55	0.117	<0.05
WS	logVA	logPHP	6.23	0.74	14	0.215	0.05
BrS	logVA	logPHP	–	–	21	–	ns
BeS	logVA	logPHP	6.41	0.81	20	0.399	<0.01

*Dp, Dependent variable; Indp Var, Independent variable. WS, Weddell Sea; BrS, Bransfield Strait; BeS, Bellingshausen Sea. Log, logarithm; ns, no significant*.

### Mortality rates of prokaryotes at the surface and in the DFM

**Figure 3:**
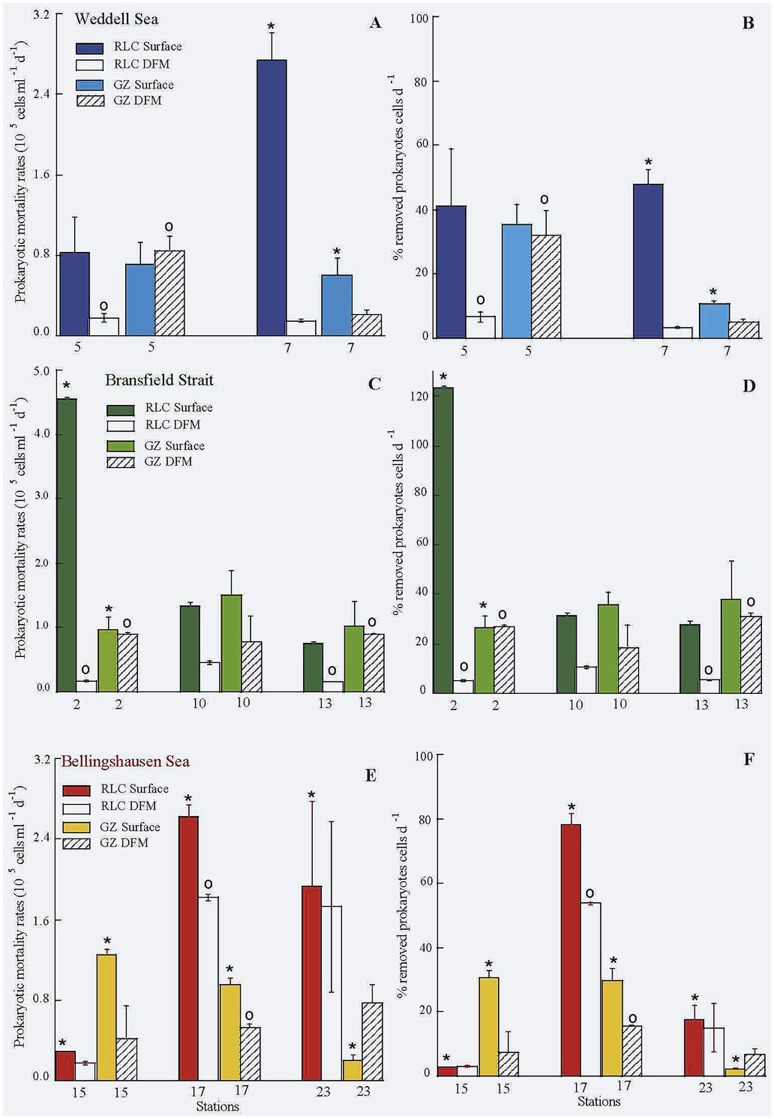
**Rate of lysed (RLC) and grazed (GZ) prokaryotes (A,C,E)**, and percentage of prokaryotic losses due to viruses and protists **(B,D,F)** in the three areas. ^*^Significant differences between viral and protists activities on prokaryotes at surface; ◦significant differences between viral and protists activities on prokaryotes at DFM.

Comparison of grazing rates of protists between surface and DFM, tended to be higher at the surface (Table 1, Table 1SM, Figure 3), except for stations 5 and 23 (Weddell and Bellingshausen Seas, respectively). But, we did not find significant differences between surface and DFM among areas (*t*-test, *n* = 16, *p* > 0.05), neither within each area. Whereas, the rate of lysed prokaryotes and percentage of lysed cells due to viruses always reached significantly higher values at the surface than in the DFM among areas (*t*-test, *n* = 16, *p* < 0.02), being significantly different within each area in the Weddell Sea (*n* = 4, *p* < 0.02), the Bransfield Strait (*n* = 6, *p* < 0.02) and in the Bellingshaussen Sea, when excluding station 23, (*n* = 4, *p* < 0.05) (Table 1, Figure 3).

Comparison between viral to protistan impact on prokaryotes at the surface showed that averages of the rate of lysed cells was always higher than grazing rates in all three areas, while in the DFM, averages of grazing rates was higher than rates of lysed cells except in the Bellingshausen Sea (Table 1, Figure 3). Looking closely to each single station, in surface, the viral impact was similar or higher than grazing rates, except at station 15 in the Bellingshausen Sea (Figures 3E–F). Significantly higher values of lysed cells were detected at stations 2, 7, 17, and 23 (Figures 3, *t*-test *n* = 2, *p* < 0.05). Conversely, the impact of grazers was similar or higher than viruses in the DFM, except at stations 17 and 23 in the Bellingshausen Sea (Figures 3E–F). Specifically, significant higher values for protistan activity in the DFM were observed at stations 2, 5, and 13 (Figure 3, *t*-test *n* = 2, *p* < 0.05).

### Top-down vs. bottom-up control

We applied the approach of Ducklow (1992), using prokaryotic biomass (PB, μg C L^−1^) plotted against prokaryotic heterotrophic production (PHP, μg C L^−1^ h^−1^) to detect whether prokaryotes were bottom-up (substrate limitation) or top-down (grazers and viruses pressure) controlled. Regarding these regressions, slopes lower than 0.4 indicate weak control by resource supply, between 0.4 and 0.6 indicate a moderate resource-limitation, and higher than 0.6 denote a stronger control by substrate. Following this convention, considering all data pooled and also the data for each of the three different areas separately, all the relationships between PB and PHP were significant and the slopes were always lower than 0.4 (all data: log PHP = 0.99 + **0.22**∗log PB, *n* = 54, *R*^2^ = 0.19, *p* < 0.001; Weddell Sea: log PHP = 1.02+ **0.37**∗log PB, *n* = 13, *R*^2^ = 0.502, *p* < 0.005; Bransfield Strait: log PHP = 0.99 + **0.17**∗log PB, *n* = 21, *R*^2^ = 0.407, *p* < 0.01; Bellingshausen Sea: log PHP = 1.15 + **0.25**∗log PB, *n* = 20, *R*^2^ = 0.179, *p* = 0.05). According to these slopes (<0.4), the prokaryotes in our area of study were top-down controlled by protists and viruses.

### Sensitivity to temperature

**Figure 4:**
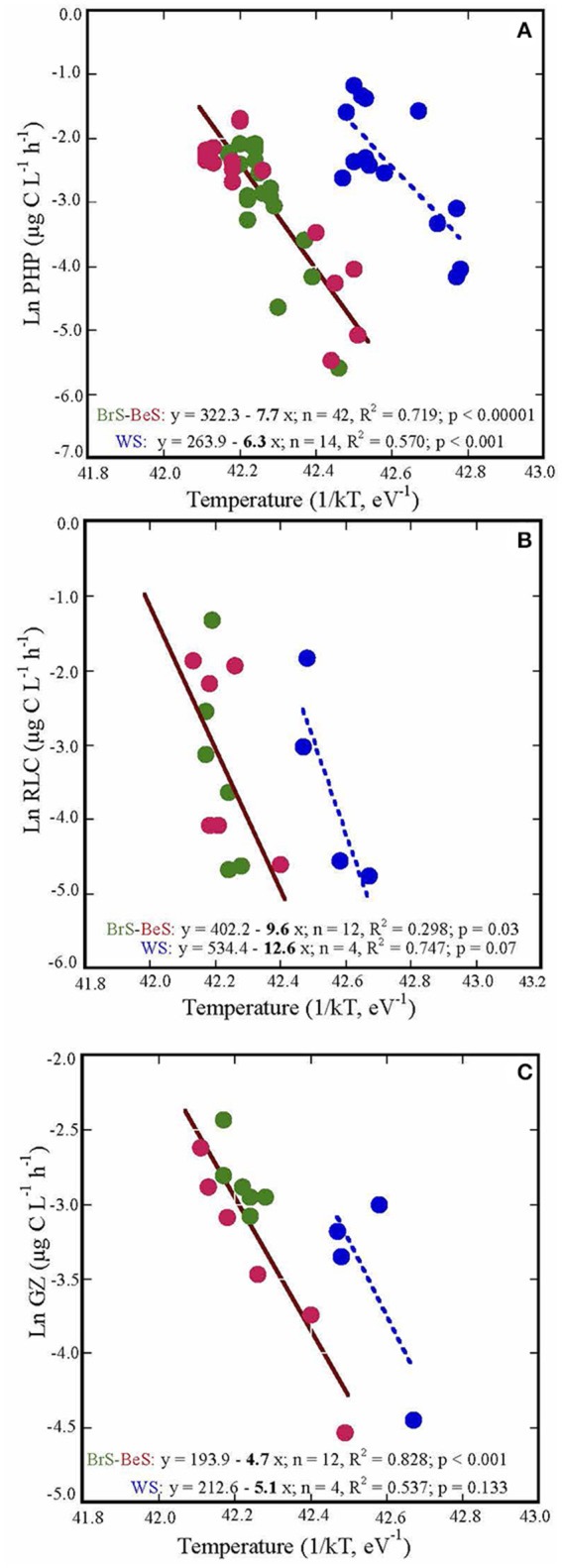
**The energy of activation (Ea), indicated by the slope coefficients of: (A)** prokaryotic heterotrophic production (PHP), **(B)** rates of lysed cells (RLC), and **(C)** grazing rates (GZ) in the three visited areas. The processes are plotted against the inverse of absolute temperature (T) multiplied by the Boltzmann constant (k) (eV^−1^). WS, Weddell Sea; BrS, Bransfield Strait; BeS, Bellingshausen Sea.

The relationships between temperature and prokaryotic heterotrophic production, grazing rates and rates of lysed cells measured in the three areas formed two groups: one that included data for the Bransfield Strait and the Bellingshausen Sea, and the other for the Weddell Sea. Both groups showed similar slopes with temperature, but different intercepts (Figure 4). PHP and the rate of lysed cells showed similar steeper slopes (Ea), 7.69 ± 0.85 and 9.60 ± 4.23, respectively, than grazing rates, 4.66 ± 0.67 (Figure 4). Consequently, PHP and RLC have higher sensitivity to temperature than grazing rates.

## Discussion

In the present study we verify our hypotheses about the impact of the predators on bacterial communities in Antarctic waters and provide new information on mortality rates of prokaryotes due to viruses and protists at surface and DFM during the Austral summer, as well as their control on prokaryotic communities, and their sensitivity to temperature.

Viral lytic production observed during this study was at the levels of the reported by Evans and Brussaard (2012) in the Weddell Sea, also during the Austral Summer, but lower than in the East sub-Antarctic zone (Evans et al., 2009). Lysogeny presented a large variation (from not detectable to 16.5 × 10^7^ virus ml^−1^ d^−1^) between the sampling points (Figure 2), as is also indicated in Evans and Brussaard (2012). Part of this variability could be attributed to the time span between sampling stations within the same region (2–10 days). In addition it is probable that we might have sampled different water masses. Noticeably, in the Weddell Sea lysogeny was very high (~80% of total viral production, Table 1), and at the same time we detected there the highest values of UF, with a phytoplanktonic community dominated by diatoms, suggesting that we could be in a situation of bloom or an initiation of bloom. This is contrary to the findings of Brum et al. (2016), who sampled a coastal area of the Western Antarctic Peninsula from November to February, and showed that in summer months (January and February) chlorophyll *a* concentration achieved high values and lysogeny was almost negligible. These authors hypothesized that lysogeny was linked to different phytoplankton bloom phases. Thus, during the initiation of the phytoplankton bloom lysogens would be induced in response to the improved trophic conditions (Stewart and Levin, 1984). This would cause the decline of lysogeny and an increase of cell lysis, which would increase the pool of dissolved organic matter. It would be also reasonable to expect that the induction of lysogens would persist along the summer and in a post-bloom situation. This would favor the availability of the pool of DOM by cells lyses together with the DOC derived from senescent phytoplankton cells, increasing prokaryotic abundance, heterotrophic production and cell lysis. Furthermore, in the Bransfield Strait and in the Bellingshausen Sea we observed low UF values, suggesting a post-bloom situation. Thus, in the Bransfield Strait, we detected lysogeny in 5 of six cases, where grazing rates achieved higher values than rates of lysed cells in the DFM (Figure 3) and similar in surface for stations 10 and 13 (Figure 3), and this was reflected by the strongest relationship between PHP and HF. In contrast, in the Bellingshausen Sea, lysogeny was only detected in the DFM at station 15, and in the other two stations we obtained high rates of lysed cells at the surface and DFM, high prokaryotic and viral abundances as well as a significant relationship between PHP and viral abundances (Table 2).

In the studied Antarctic waters, mortality rates of prokaryotes due to viral activity achieved the maximum values, and significantly higher than those by protistan grazing in several surface samples (Figure 3). These results are in agreement to those in Guixa-Boixereu et al. (2002) where viral activity was higher than grazing by protists in the Bransfield Strait, the Gerlache Strait and the Bellingshaussen Sea. In contrast, a similar or higher impact of grazers than viruses was found in the DFM. A possible explanation is that HF and mainly PF abundances were similar or higher in the DFM than in the surface (Table 1SM). It might be likely that part of the PF considered in this study as phototrophic microorganisms, might be mixotrophs, which is a common feature in Antarctic waters, representing 10% of all chloroplastidic nanoflagellates in the water column (Moorthi et al., 2009).

As a result of viral and protist activities, during the Austral summer prokaryotes were top-down controlled according to the approach of Ducklow (1992). This is corroborated by the fact that both predators removed on average a high percentage of prokaryotic biomass (46.60 ± 9.24% d^−1^) and production (116.09 ± 13.01% d^−1^) and these losses would not be sustainable if there was not enough DOC to maintain the growth of the population. Brum et al. (2016) and Evans and Brussaard (2012) stated that the release of DOM from prokaryote lysates in the onset of blooms could also contribute to maintain the sufficient levels of substrate for prokaryotic growth. Also Morán and Estrada (2002) in the Bransfield Strait measured the supply of photosynthate by phytoplankton, showing that the DOC released by primary producers overcome the prokaryotic requirements, while Guixa-Boixereu et al. (2002) in the same cruise reported high mortality rates on prokaryotes, corroborating the top-down control. In contrast, in Franklin Bay in the Arctic Ocean prokaryotic community in spring was bottom-up regulated, suggesting that prokaryotic growth was controlled only by substrate and escaping predation (Vaqué et al., 2008).

In order to summarize in a big picture the main fluxes of carbon in the studied Antarctic areas (notice that respiration was not measured in this study), we performed a rough estimation of the carbon biomass and fluxes (PHP, rate of lysed cells and grazing) averaging all values from each area (Table 3). According to that, the viral shunt of prokaryotic carbon to the water column provided as much as 1.4 ± 0.5 μg C L^−1^ d^−1^ in the Weddell Sea, 1.7 ± 1.0 μg C L^−1^ d^−1^ in the Bransfield Strait, and 2.0 ± 0.6 μg C L^−1^ d^−1^ in the Bellingshausen Sea. At the same time, the carbon ingested by small bacterivores, HF ≤ 5 μm, was similar or lower to that released due to viral lysis (0.8 ± 0.2 μg C L^−1^ d^−1^ in the Weddell Sea, 1.4 ± 0.1 μg C L^−1^ d^−1^ in the Bransfield Strait and 0.9 ± 0.2 in the Bellingshausen Sea), while PHP values ranged from 3.4 ± 0.9 μg C L^−1^ d^−1^ in the Weddell Sea to 1.9 ± 0.2 μg C L^−1^ d^−1^ in the Bellingshausen Sea. In terms of carbon biomass, average prokaryotic carbon was considerably higher (10.6 ± 2.2 μg C L^−1^) and the carbon biomass of bacterivorous HFB ≤ 5 μm was the lowest (2.7 ± 0.5 μg C L^−1^) in the Bellingshausen Sea (Table 3). This global estimation suggests a significant contribution of viral activity in the Austral Summer and agrees with the results obtained by Christaki et al. (2014) in the Kergelen Area (Sub-Antarctic ocean). These authors found that the absolute amount of bacterial carbon channeled toward the higher trophic levels was the same in spring and summer, but the underlying mechanisms were contrasting. In spring, the PHP was relatively low and most of it was channeled to the higher trophic levels due to predation, while in summer, the PHP was higher, but most of it was returned to the dissolved phase through viral lysis. It is plausible that higher lysis in summer might denote the result of the induction of lysogens, agreeing with the findings of Brum et al. (2016) that viral activity was more important during the Austral Summer, when the phytoplankton bloom prevails.

**Table 3:** **Biomass of prokaryotes (PB), viruses (VB), heterotrophic pico/nanoflagellates (HFB ≤5 μm), phototrophic pico/nanoflagellates (PFB), prokaryotic heterotrophic production (PHP ± SD), mortality rates due to grazers (GZ ± SD), and to viral lyses (RLC ± SD) at the surface and deep fluorescence maximum (DFM)**.

**Site**	**St**	**Date**	**Z**	**PB**	**VB**	**HFB_≤5_**	**PFB**	**PHP**	**GZ**	**RLC**
**WS**	5	01-Feb	0.1	2.82	0.05	2.27	18.64	1.75 ± 0.31	1.00 ± 0.31	1.16 ± 0.50
	5	01-Feb	35	3.73	0.06	2.73	29.34	1.90 ± 0.08	1.20 ± 0.19	0.25 ± 0.05
	7	03-Feb	0.1	8.10	2.74	5.99	13.40	4.91 ± 0.28	0.84 ± 0.25	3.87 ± 0.38
	7	03-Feb	20	6.30	1.96	13.87	24.84	4.98 ± 0.64	0.28 ± 0.08	0.21 ± 0.02
				**5.2 ± 1.2**	**1.5 ± 0.5**	**6.2 ± 2.3**	**19.1 ± 2.0**	**3.41 ± 0.30**	**0.83 ± 0.17**	**1.37 ± 0.45**
**BrS**	2	29-Jan	0.1	5.19	0.23	0.30	3.78	0.92 ± 0.14	1.35 ± 0.28	6.42 ± 0.04
	2	29-Jan	30	4.67	0.30	4.45	68.92	1.51 ± 0.03	1.25 ± 0.06	0.24 ± 0.02
	10	06-Feb	0.1	5.99	0.79	4.37	20.45	2.55 ± 0.34	2.11 ± 0.55	1.87 ± 0.08
	10	06-Feb	30	6.04	2.03	2.43	23.09	2.76 ± 0.14	1.10 ± 0.57	0.64 ± 0.03
	13	09-Feb	0.1	3.83	0.25	5.13	22.47	2.60 ± 0.35	1.45 ± 0.53	1.06 ± 0.05
	13	09-Feb	15	4.11	0.18	3.08	27.90	2.30 ± 0.13	1.26 ± 0.02	0.22 ± 0.01
				**5.0 ± 0.4**	**0.6 ± 0.3**	**3.3 ± 0.7**	**27.8 ± 8.1**	**2.11 ± 0.19**	**1.42 ± 0.36**	**1.74 ± 0.97**
**BeS**	15	11-Feb	1	14.7	0,10	2.03	12.60	2.34 ± 0.16	1.74 ± 0.09	0.41 ± 0.00
	15	11-Feb	55	7.65	0,10	1.10	35.91	0.75 ± 0.04	0.57 ± 0.48	0.24 ± 0.02
	17	13-Feb	0.1	4.73	0.39	2.51	7.37	2.25 ± 0.14	1.35 ± 0.08	3.71 ± 0.17
	17	13-Feb	25	4.77	1.16	2.67	12.11	1.99 ± 0.30	0.74 ± 0.05	2.56 ± 0.04
	23	20-Feb	1	15.4	0.79	3.26	73.03	2.27 ± 0.20	0.26 ± 0.09	2.73 ± 1.19
	23	20-Feb	25	16.1	0.45	4.59	77.94	2.01 ± 0.15	1.09 ± 0.25	2.44 ± 1.20
				**10.5 ± 2.2**	**0.5 ± 0.2**	**2.7 ± 0.5**	**36.5 ± 13.0**	**1.93 ± 17**	**0.95 ± 0.22**	**2.02 ± 0.56**

*Biomasses are expressed in μg C L^−^^1^ and rates in μg C L^−^^1^ d^−^^1^. WS, Weddell Sea; BrS, Bransfield Strait; BeS, Bellingshausen Sea. St, station; Z, depth (surface and DFM). In bold averaged values ± SE*.

Temperature played an important role in the studied microbial processes (PHP, GZ and RLC) and PHP and RLC responded quicker to small changes than grazing rates (Figure 4). In contrast, Maranger et al. (2015) showed that PHP and GZ were more sensitive to temperature than viral activity in Arctic regions. This could be due to the fact that in the Arctic, during the summer season, the range of temperature is wider (−1.87 to 9.0, Maranger et al., 2015) than in Antarctic waters (−1.87 to 4.0, Danovaro et al., 2011). Thus, Arctic HFs were probably well adapted to this wide range, and responded quickly to small increases of temperature. In contrast, HFs in Antarctic waters are used to live in a narrower range of cold temperatures and do not respond as quickly when the temperature increases (Vaqué et al., 2009). Furthermore, Sarmento et al. (2010) in a study considering data of PHP and GZ from temperate to Antarctic systems also found that PHP (Ea, slope = 0.67) was more sensitive to temperature than GZ (Ea, slope = 0.47), although they did not have viral activities to compare with. Indeed, our results suggest a decrease of the fraction of PHP taken by protists and an increase of that recirculating through viral lysis in a warmer ocean. Such a change in the carbon fluxes would imply lower microbial carbon efficiency in the channeling of organic matter to higher trophic levels, and increase of respiration. Together with the few studies on the different sensitivity of these two mortality processes, our work may help to predict the destiny of the microbial carbon in a warmer ocean. However, it may be necessary to carry out more studies on the effect of temperature on microbial processes in order to confirm our empirical observations.

## Conclusions

Our results indicate that grazers and viruses controlled the prokaryotic community in the visited Antarctic areas during the Austral summer 2009. Viruses had the highest impact on prokaryotes mainly at the surface, while protists activity played an important role in the DFM. In the big picture, the contribution of the prokaryotic carbon fuelled to the water column due to viral activity was higher than the prokaryotic carbon transferred to higher trophic levels by grazers. Prokaryotic heterotrophic production and viral activity were more sensitive to temperature than protists grazing, suggesting a lower flux of C through the microbial loop. This fact should be confirmed in further field and experimental studies in Antarctic waters to understand future changes in microbial dynamics in a warmer ocean scenario.

## Author contributions

DV and JB designed the study, collected samples for viruses, prokaryotes and protists, performed mortality experiments, analyzed the results and wrote the manuscript; SA coordinated the phytoplankton sampling, its analysis and identification, and provided constructive criticisms to the manuscript; FT, YC, and EL processed the abundances of viral and protists profiles, as well as viral and prokaryotic abundances in experimental samples, and also helped with data analysis; JA performed heterotrophic prokaryotic production and prokaryotic abundances in natural samples, adding clever comments to the text; CD coordinator of the ATOS project, provided a creative environment and added constructive criticisms throughout the study; MS provided constructive comments, revised and edited the manuscript. All authors commented and discussed the obtained results, and suggested improvements on the manuscript.

## Funding

This study was supported by the following projects: ATOS (POL2006-00550/CTM, P.I.: CD.) funded by the Spanish Ministerio de Ciencia e Innovación, YC work was supported by a Ph.D. fellowship from the MINECO (FPI grant).

### Conflict of interest statement

The authors declare that the research was conducted in the absence of any commercial or financial relationships that could be construed as a potential conflict of interest.
