# Supplementary material for: Viruses and Protists Induced-mortality of Prokaryotes around the Antarctic Peninsula during the Austral Summer
Source: Front Microbiol. 2017 Mar 2;8:241. doi: 10.3389/fmicb.2017.00241 (PMC5332362; doi:10.3389/fmicb.2017.00241)
Supplement: Supplementary file 1 [file Table1.pdf]

Table 1SM. Location, stations (St), date, situation (latitude S, longitude W), depth (m), temperature (°C), salinity, Units of Fluorescence (UF), pico/nanophototrophic flagellates (PF, 10^3 cel ml^{-1}) Prokaryotic and viral abundance (PA, 10^5 cel ml^{-1} and VA, 10^6 vir ml^{-1}), prokaryotic heterotrophic production (PHP, µg C L^{-1}d^{-1}), pico/nanoheterotrophic flagellates (HF, 10^3 cel ml^{-1}), viral lytic production (VP_L, 10^7 vir ml^{-1}d^{-1}), Lysogeny (VP_{Lyso} 10^7 vir ml^{-1}d^{-1}), and rates of lysed cells (RLC, 10^4 cel ml^{-1} d^{-1}), and grazing rates (GZ, 10^4 cel ml^{-1} d^{-1}). nd: not detected, -: no data. In bold: depth corresponding to Deep Fluorescence Maximum (DFM). Grey areas correspond to the stations where mortality rates were measured. SE: standard error, SD: standard deviation of duplicates.

Site	St	Date	Lat	Long	Depth	T	S	UF	PF	PA	VA	PHP±SE	HF	VP_L±SD	VP_{Lyso}±SD	RLC±SD	GZ±SD
BrS	1	28/01	62°39.02	59°00.11	0.1	2.4	30.3	-	-	3.7	3.7	2.5±0.2	-	-	-	-	-
					5	2.1	30.3	2.4	-	3.4	5.6	2.3±0.6	-	-	-	-	-
					10	2.1	30.3	2.7	-	3.2	2.4	2.4±0.7	-	-	-	-	-
					**20**	2.1	30.2	2.9	-	3.1	2.7	1.7±0.6	-	-	-	-	-
					40	1.9	28.9	2.2	-	3.1	4.8	1.9±0.2	-	-	-	-	-
					70	0.4	30.3	0.2	-	4.1	6.2	0.4±0.3	-	-	-	-	-
Brs	**2**	29/01	61°59.59	55°40.54	0.1	1.8	-	-	0.5	3.7	2.3	0.9±0.1	0.3	4.4±0.03	nd	45.5±0.29	9.6±2.01
					1	-	-	-	3.8	4.2	2.2	1.2±0.0	0.7	-	-	-	-
					5	-	-	-	4.4	3.7	1.3	1.3±0.1	0.8	-	-	-	-
					15	1.4	29.7	1.7	4.2	3.4	7.2	1.3±0.1	0.9	-	-	-	-
					**30**	1.4	29.7	2.0	9.4	3.3	3.0	1.5±0.0	1.8	0.2±0.02	0.03±0.02	1.7±0.15	8.9±0.39
					50	1.3	29.6	1.6	4.1	3.9	2.6	1.1±0.2	0.7	-	-	-	-
					80	0.8	29.2	0.5	1.5	3.6	2.7	0.7±0.1	0.6	-	-	-	-
WS	3	30/01	61°40.57	52°30.01	0.1	1.0	-	-	-	3.5	1.5	2.2±0.2	-	-	-	-	-
					1	0.7	-	-	-	3.7	2.2	1.8±0.9	-	-	-	-	-
					5	0.5	28.9	0.4	-	2.9	1.3	2.3±0.0	-	-	-	-	-
					15	0.5	28.9	0.5	-	3.7	0.7	2.1±0.1	-	-	-	-	-
					**35**	0,3	28.8	1.0	-	3.1	3.4	2.2±0.2	-	-	-	-	-
					50	0.2	28.8	0.9	-	3.0	4.5	1.0±0.1	-	-	-	-	-
					80	0.1	28.7	0.7	-	3.3	3.5	1.0±0.0	-	-	-	-	-
WS	4	31/01	62°40.04	51°29.26	1	0.4	-	-	-	4.3	2.7	1.9±0.2	-	-	-	-	-
					5	0.4	-	-	-	2.4	1.2	2.4±0.6	-	-	-	-	-
					15	-0.1	27.9	0.4	-	2.3	2.2	1.9±0.9	-	-	-	-	-
					**33**	-0.1	27.6	0.9	-	3.6	1.3	2.3±0.9	-	-	-	-	-
					55	-1.4	27.4	0.4	-	3.2	0.7	0.4±0.1	-	-	-	-	-
					80	-1.6	27.3	0.3	-	1.3	2.3	0.1±0.0	-	-	-	-	-
WS	**5**	01/02	63°51.49	51° 51.47	0.1	0.1	-	-	2.5	2.0	0.5	1.8±0.3	1.0	1.0±0.2	nd	8.2±3.53	7.1±2.16
					1	-0.1	-	-	3.1	1.8	0.7	2.3±0.2	0.8	-	-	-	-
					5	-0.3	-	0.5	3.0	1.8	5.1	2.4±0.1	0.7	-	-	-	-
					20	-0.3	27.7	0.8	3.9	2.9	1.7	2.1±0.2	1.0	-	-	-	-
					**35**	-0.5	27.7	0.8	4.0	2.6	0.6	1.9±0.1	1.2	0.2±0.05	0.8±0.00	1.8±0.39	8.5±1.37
					60	-1.5	27.6	0.7	3.2	4.1	11.3	0.9±0.0	0.9	-	-	-	-
					80	-1.7	27.2	0.4	1.2	2.4	3.2	0.4±0.0	0.5	-	-	-	-
WS	6	02/02	63°55.46	53°52.32	0.1	-0.4	-	-	-	2.0	3.4	2.8±0.5	-	-	-	-	-
					1		-	-	-	1.9	1.8	1.8±0.0	-	-	-	-	-
					5	-0.8	-	0.6	-	3.0	7.0	4.6±0.6	-	-	-	-	-
					**20**	-1.0	27.4	0.9	-	2.4	2.6	4.6±1.1	-	-	-	-	-

Site	St	Date	Lat	Long	Depth	T	S	UF	PF	PA	VA	PHP±SE	HF	VP_L±SD	VP_{Lyso}±SD	RLC±SD	GZ±SD
WS	6	02/02	63°55.46	53°52.32	30	-1.1	27.5	0.7	-	4.1	5.4	4.8±0.4	-	-	-	-	-
					50	-1.4	27.5	0.6	-	3.8	0.2	2.8±1.0	-	-	-	-	-
					80	-1.7	27.4	0.5	-	2.0	0.2	0.4±0.0	-	-	-	-	-
WS	**7**	03/02	65°01.10	55°45.26	0.1	0.1	-	-	1.8	5.7	27.4	4.9±0.3	2.7	3.5±0.34	16.5±0.00	27.4±0.27	6.0±1.76
					1	0.0	-	-	3.5	6.3		7.5±0.3	3.0				
					5	-0.2	27.9	14.6	5.3	5.4	22.1	6.3±0.7	1.5				
					10	-0.2	27.9	10.5	2.7	5.6	2.0	6.2±0.1	1.5				
					**20**	-1.1	27.4	14.4	3.4	4.5	19.6	5.0±0.6	5.1	1.9±0.02	nd	1.5±0.13	2.0±0.16
					40	-1.8	27.1	0.7	1.5	2.1	0.6	1.1±0.1	3.0				
					80	-1.8	27.1	0.2	1.1	1.6	0.6	0.4±0.0	1.6				
WS	8	04/02	64°16.11	55°51.31	0.1	0.1	-	-	-	4.4	4.5	3.2±0.4	-	-	-	-	-
					1	0.0	-	-	-	3.7	2.2	4.8±0.4	-	-	-	-	-
					5	-0,2	28.4	6.7	-	3.3	1.3	4.5±1.0	-	-	-	-	-
					15	-0.2	28.4	6.4	-	3.6	0.7	3.7±1.1	-	-	-	-	-
					**25**	-0.2	28.4	6.0	-	4.1	11.4	4.9±0.5	-	-	-	-	-
					40	-0.3	28.3	3.7	-	3.4	12.1	2.6±0.5	-	-	-	-	-
					80	-0.7	28.0	0.7	-	3.6	4.7	1.2±0.3	-	-	-	-	-
WS	9	05/02	63°26.20	56°39.07	0.1	0.0	-	-	-	3.3	1.3	1,6±0.1	-	-	-	-	-
					1	-0.2	-	-	-	2.6	3.0	0.9±0.1	-	-	-	-	-
					5	-0.4	28.3	0.4	-	2.7	0.6	1.1±0.7	-	-	-	-	-
					30	-0.3	28.3	0.9	-	2.7	1.6	1.4±0.1	-	-	-	-	-
					**50**	-0.4	28.3	1.2	-	2.6	1.2	1.4±0.2	-	-	-	-	-
					70	-0.4	28.3	1.3	-	3.1	0.3	1.5±0.1	-	-	-	-	-
					90	-0.5	28.2	0.7	-	2.6	0.9	1.2±0.0	-	-	-	-	-
BrS	**10**	06/02	62°10.38	57°14.25	0.1	2.1	-	-	2.8	4.2	7.9	2.6±0.3	1.7	1.7±0.07	0.2±0.02	13.3±0.55	14.9±3.89
					1	1.9	-	-	3.0	4.6	2.2	3.0±0.1	0.8	-	-	-	-
					5	1.7	30.0	2.0	2.0	4.7	1.3	2.9±0.2	1.3	-	-	-	-
					15	1.7	29.9	2.3	2.2	4.2	0.7	3.0±0.2	1.7	-	-	-	-
					**30**	1.7	29.9	2.2	3.1	4.3	20.3	2.8±0.1	1.1	0.6±0.03	0.04±0.02	4.5±0.25	7.81±4.02
					50	1.6	28.9	1.7	1.6	3.8	9.7	1.9±0.1	0.9	-	-	-	-
					70	0.7	29.2	0.4	0.9	3.0	5.0	0.4±0.0	0.3	-	-	-	-
BrS	11	07/02	62°25.28	58°10.05	1	-	-	-	-	5.9	12.9	2.2±0.3	-	-	-	-	-
					5	1.9	30.2	0.8	-	6.4	15.7	1.9±0.0	-	-	-	-	-
					15	1.9	30.1	2.9	-	6.2	2.2	2.2±0.2	-	-	-	-	-
					**30**	1.8	30.1	3.9	-	5.8	1.3	1.9±0.3	-	-	-	-	-
					50	1.7	30.0	2.9	-	6.4	0.7	2.1±0.3	-	-	-	-	-
					70	0.5	28.9	0.5	-	2.6	6.0	0.3±0.0	-	-	-	-	-
BrS	12	08/02	62°38.59	60°24.26	0.1	2.8	-	-	-	6.5	17.9	4.5±0.1	-	-	-	-	-
					1	-	-	-	-	6.5	2.5	4.0±0.3	-	-	-	-	-
					8	-	-	-	-	5.8	5.4	4.3±0.4	-	-	-	-	-
					15	2.4	30.4	2.3	-	2.0	17.9	4.1±0.2	-	-	-	-	-
					**20**	2.2	30.3	2.1	-	1.7	6.9	3.1±0.1	-	-	-	-	-
					40	2.1	30.2	1.4	-	3.1	8.7	1.5±0.2	-	-	-	-	-

Site	St	Date	Lat	Long	Depth	T	S	UF	PF	PA	VA	PHP±SE	HF	VP_L±SD	VP_{Lvso}±SD	RLC±SD	GZ±SD
BrS	12	08/02	62°38.59	60°24.26	70	2.0	30.1	1.1		4.6	6.9	1.4±0.0		-			
BrS	**13**	09/02	63°33.58	61°16.06	0.1	2.1	-	-	3.1	6.5	2.5	2.6±0.4	2.5	0.7±0.03	0.3±0.18	7.5±0.35	10.2±3.78
					1	1.9	-	-	3.6	6.5	4.6	2.2±0.3	2.1	-	-	-	-
					5	1.7	29.9	3.1	6.3	5.8	6.0	2.3±0.1	2.6	-	-	-	-
					15	1.7	29.9	2.8	3.8	2.0	1.8	2.3±0.1	1.9	-	-	-	-
					**30**	1.5	29.7	2.2	5.0	1.7	0.9	1.4±0.1	1.7	0.1±0.00	0.1±0.05	1.6±0.04	8.9±0.12
					40	1.2	29.5	1.7	1.7	3.1	4.7	0.2±0.1	0.4	-	-	-	-
					70	0.3	28.8	0.2	1.4	4.6	2.5	0.1±0.1	0.3	-	-	-	-
BeS	**15**	11/02	63°40.00	62°54.59	1	-	-	-	1.7	10.4	1.1	2.3±0.2	0.8	0.4±0.00	nd	2.9±0.00	12.4±0.65
					5	1.3	30.4	0.5	3.0	6.6	1.2	2.6±0.1	0.7	-	-	-	-
					25	1.3	30.4	0.6	2.2	7.4	1.2	2.7±0.1	0.3	-	-	-	-
					**55**	1.3	28.9	1.3	4.9	5.4	1.0	0.8±0.0	0.4	0.2±0.05	0.3±0.03	1.7±0.17	4.04±3.43
					70	1.3	28.7	0.9	2.2	3.4	0.6	0.3±0.0	0.3	-	-	-	-
					100	1.1	28.8	0.3	6.4	2.0	0.2	0.1±0.0	0.2	-	-	-	-
BeS	16	12/02	65°03.14	66°56.25	0.1	3.2	-	-	-	7.4	15.9	3.1±0.3	-	-	-	-	-
					1	3.1	-	-	-	7.2	2.3	1.9±0.3	-	-	-	-	-
					5	2.9	30.6	0.3	-	7.5	2.8	2.2±0.0	-	-	-	-	-
					25	2.5	30.3	0.4	-	7.7	1.8	2.7±0.1	-	-	-	-	-
					**50**	0.4	28.6	0.7	-	6.2	1.2	1.8±0.3	-	-	-	-	-
					70	-0.2	28.2	0.5	-	3.1	0.5	0.1±0.0	-	-	-	-	-
					100	0.0	28.6	0.2	-	1.7	0.3	0.5±0.0	-	-	-	-	-
BeS	**17**	13/02	67°24.33	70°50.20	0.1	2.3	-		1.0	3.4	3.4	2.3±0.1	1.0	0.3±0.01	nd	26.2±1.20	9.5±0.59
					1	-	-		0.7	2.3	10.0	2.8±0.3	1.1	-	-	-	-
					5	-	-		1.5	2.5	7.2	2.2±0.8	1.4	-	-	-	-
					10	1.9	29.7	0.3	1.3	3.4	17.2	4.3±0.1	1.4	-	-	-	-
					15	1.9	29.7	0.5	1.9	2.9	60.6	4.5±0.3	1.8	-	-	-	-
					**25**	1.5	29.4	1.4	1.6	3.4	11.6	2.0±0.3	1.1	2.3±0.04	nd	18.2±0.30	5.3±0.35
					60	2.3	28.3	0.2	0.5	3.7	11.7	0.4±0.1	0.3	-	-	-	-
BeS	18	14/02	69°02.35	75°06.15	0,1	-	-	-	-	3.7	16.3	3.6±0.2	-	-	-	-	-
					1	-	-	-	-	5.0	7.9	2.0±0.3	-	-	-	-	-
					5	1.0	28.8	0.7	-	4.2	3.9	2.3±0.1	-	-	-	-	-
					**12**	0.8	28.7	0.7	-	4.0	6.5	3.3±0.1	-	-	-	-	-
					20	-0.1	27.9	0.4	-	4.6	12.3	1.7±0.1	-	-	-	-	-
					40	-0.9	27.3	0.3	-	4.1	2.0	0.6±0.0	-	-	-	-	-
					60	-0.1	28.1	0.3	-	4.6	11.4	0.7±0.1	-	-	-	-	-
BeS	19	15/02	69°33.09	74°31.28	0,1	-	-	-	-	2.4	35.6	4.0±0.4	-	-	-	-	-
					1	-	-	-	-	2.2	83.1	3.7±0.6	-	-	-	-	-
					5	-1.5	26.2	3.3	-	2.5	25.2	3.3±0.5	-	-	-	-	-
					**12**	-1.0	26.8	2.6	-	3.3	14.0	2.7±0.2	-	-	-	-	-
					20	-0.4	27.4	1.1	-	3.8	8.6	2.5±0.1	-	-	-	-	-
					30	0.4	28.3	1.4	-	3.6	5.3	3.4±0.2	-	-	-	-	-
					50	0.1	28.1	0.6	-	4.4	35.6	1.7±0.5	-	-	-	-	-
BeS	22	19/02	67°22.15	69°48.22	0,1	2.0	-	-	-	5.0	6.7	2.2±0.1	-	-	-	-	-

Site	St	Date	Lat	Long	Depth	T	S	UF	PF	PA	VA	PHP±SE	HF	VP_L±SD	VP_{Lyso}±SD	RLC±SD	GZ±SD
BeS	22	19/02	67°22.15	69°48.22	1	1.7	-	-	-	4.6	5.7	1.4±0.2	-	-	-	-	-
					5	1.4	27.0	0.9	-	5.1	5.4	1.5±0.1	-	-	-	-	-
					10	1.6	29.5	1.3	-	5.1	13.3	1.6±0.4	-	-	-	-	-
					**25**	1.6	29.5	1.4	-	5.0	8.0	2.1±0.2	-	-	-	-	-
					40	0.2	28.5	0.4	-	4.9	9.0	2.0±0.1	-	-	-	-	-
					70	-0.4	28.1	0.2	-	2.1	2.8	0.3±0.0	-	-	-	-	-
BeS	**23**	20/02	66°01.59	67°10.03	1	-	-	-	9.9	10.9	7.9	2.3±0.2	1.2	1.5±0.66	nd	19.3±8.46	1.8±0.66
					5	2.1	29.9	1.4	10.4	11.2	2.3	2.2±0.1	1.9	-	-	-	-
					10	2.1	29.9	1.3	9.1	11.4	4.6	2.3±0.2	1.6	-	-	-	-
					**25**	2.1	29.9	1.6	10.6	11.4	4.5	2.0±0.2	1.9	1.4±0.07	nd	17.3±8.48	7.7±1.78
					40	2.0	29.9	1.4	7.6	9.4	3.3	1.6±0.3	0.7	-	-	-	-
					70	-0.1	28.3	0.3	1.3	1.9	1.3	0.2±0.0	0.2	-	-	-	-
BeS	24	21/02	64°56.28	64°32.14	0,1	2.0	-	-	-	3.1	1.3	3.2±0.5	-	-	-	-	-
					1	2.2	-	-	-	3.4	8.8	3.5±0.3	-	-	-	-	-
					5	2.5	30.3	4.1	-	2.9	5.2	3.2±0.2	-	-	-	-	-
					**15**	2.4	30.3	4.9	-	3.8	20.0	3.0±0.3	-	-	-	-	-
					25	2.3	30.2	2.6	-	3.7	15.4	2.5±0.0	-	-	-	-	-
					32	2.3	30.2	1.6	-	3.7	45.5	2.5±0.1	-	-	-	-	-
					50	1.5	29.8	0.2	-	3.2	43.2	0.7±0.0	-	-	-	-	-
BrS	26	23/02	63°02.35	57°32.07	0,1	-0.5	-	-	-	2.6	2.5	0.8±0.1	-	-	-	-	-
					1	-0.5	-	-	-	2.3	7.7	1.2±0.2	-	-	-	-	-
					5	-0.6	28.1	0.5	-	2.3	8.6	1.0±0.1	-	-	-	-	-
					20	-0.6	28.1	0,6	-	2.5	6.2	1.1±0.2	-	-	-	-	-
					**30**	-0.6	28.1	0,6	-	2.2	14.9	0.8±0.1	-	-	-	-	-
					55	-0.6	28.1	0,5	-	2.2	9.1	0.8±0.1	-	-	-	-	-
					80	-0.8	28.0	0,3	-	1.8	2.9	0.1±0.1	-	-	-	-	-
WS	27	24/02	63°05.08	54°46.14	1	-0.6	27.6	0.6	-	2.0	8.1	1.6±0.2	-	-	-	-	-
					5	-0.6	28.1	1.0	-	1.6	5.1	1.6±0.2	-	-	-	-	-
					15	-0.6	28.1	1.3	-	1.5	5.7	1.3±0.1	-	-	-	-	-
					**30**	-0.6	28.1	2.0	-	1.5	8.7	1.4±0.1	-	-	-	-	-
					50	-0.5	28.1	1.6	-	1.6	10.4	1.3±0.4	-	-	-	-	-
					80	-0.5	28.1	1.3	-	1.6	6.6	1.7±0.3	-	-	-	-	-
WS	28	25/02	64°57.01	55°50.00	0,1	-0.8	-	-	-	5.4	3.0	9.5±0.8	-	-	-	-	-
					1	-0.8	-	-	-	-	4.5	6.3±0.8	-	-	-	-	-
					5	-0.7	27.6	0.4	-	-	2.1	7.0±0.3	-	-	-	-	-
					18	-0.8	27.6	1.5	-	-	2.7	6.2±0.5	-	-	-	-	-
					**35**	-1.5	27.3	2.0	-	-	0.3	4.7±0.2	-	-	-	-	-
					45	-1.7	27.2	1.0	-	-	0.5	2.3±0.3	-	-	-	-	-
					60	-1.8	27.2	0.3	-	-	0.2	0.7±0.1	-	-	-	-	-
