# Supplementary material for: Viruses and Protists Induced-mortality of Prokaryotes around the Antarctic Peninsula during the Austral Summer
Source: Front Microbiol. 2017 Mar 2;8:241. doi: 10.3389/fmicb.2017.00241 (PMC5332362; doi:10.3389/fmicb.2017.00241)
Supplement: Supplementary file 2 [file Table2.PDF]

Table 2SM. Results of the ANOVA test for physicochemical and biological variables that are significantly different among the whole three areas in bold (**All**). When the ANOVA test was significant, differences between each two areas were assessed with Post hocTukey tests. Significant values are indicated with p<0.05. ns: no significant, n = number of data, F: Fisher value, p: level of significance. Variables that does not present significant differences are not included.

**Variables**		**n**	**F**	**p**
**Temperature**	**All**	**150**	**59.79**	**<0.0001**
	Weddell-Bransfield	102	115.14	<0.0001
	Bransfield-Bellingshausen	90	0.07	ns
	Bellingshausen-Weddell	108	88.37	<0.0001
**Salinity**	**All**	**117**	**40.95**	**<0.0001**
	Weddell-Bransfield	78	132.44	<0.0001
	Bransfield-Bellingshausen	78	6.36	<0.02
	Bellingshausen-Weddell	84	33.2	<0.0001
**UF**	**All**	**117**	**3.45**	**<0.05**
	Weddell-Bransfield	78	0.77	ns
	Bransfield-Bellingshausen	72	5.22	<0.03
	Bellingshausen-Weddell	84	2.96	ns
**Prok. ab**	**All**	**154**	**12.76**	**<0.0001**
	Weddell-Bransfield	100	5.08	0.026
	Bransfield-Bellingshausen	99	7.04	<0.01
	Bellingshausen-Weddell	109	23.09	<0.001
**Viral ab.**	**All**	**160**	**9.68**	**<0.0001**
	Weddell-Bransfield	107	13.56	<0.001
	Bransfield-Bellingshausen	100	0.20	ns
	Bellingshausen-Weddell	113	13.87	<0.001
**HF ab.**	**All**	**54**	**3.09**	**0.05**
	Weddell-Bransfield	35	2.71	ns
	Bransfield-Bellingshausen	40	1.05	ns
	Bellingshausen-Weddell	33	5.97	<0.05
**GZ**	**All**	**16**	**5.83**	**<0.05**
	Weddell-Bransfield	10	5.24	0.05
	Bransfield-Bellingshausen	12	3.30	0.05
	Bellingshausen-Weddell	10	0.06	ns
**% PSS_{GZ}**	**All**	**16**	**2.99**	**0.05**
	Weddell-Bransfield	10	0.83	ns
	Bransfield-Bellingshausen	12	9.77	0.02
	Bellingshausen-Weddell	10	1.23	ns
