# Supplementary figures and images for: Viruses and Protists Induced-mortality of Prokaryotes around the Antarctic Peninsula during the Austral Summer
Source: Front Microbiol. 2017 Mar 2;8:241. doi: 10.3389/fmicb.2017.00241 (PMC5332362; doi:10.3389/fmicb.2017.00241)

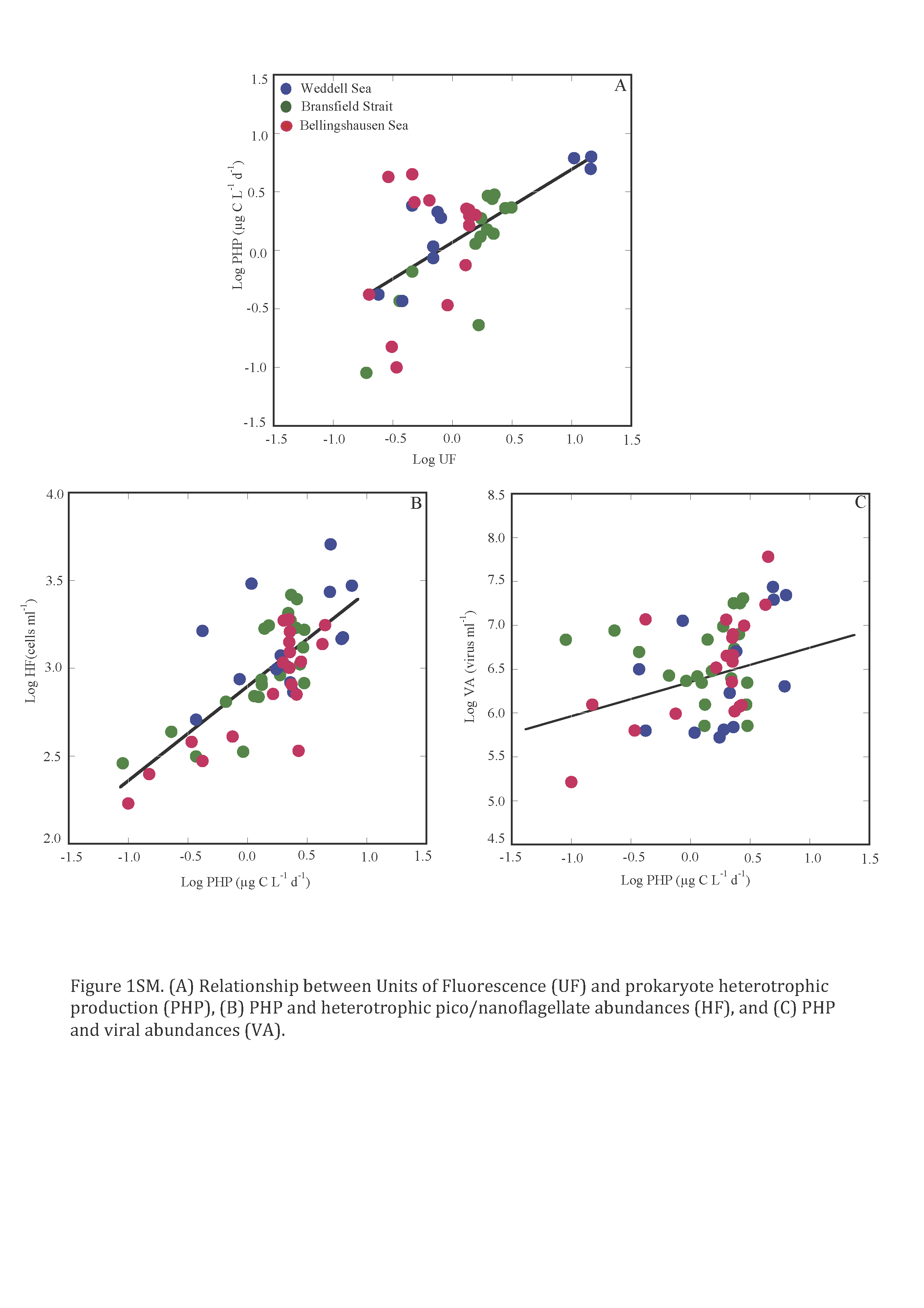

Supplement: Supplementary file 3 [file Image1.TIFF]
